# It takes three to tango: An ethnography of triadic involvement of residents, families and nurses in long‐term dementia care

**DOI:** 10.1111/hex.13224

**Published:** 2021-07-20

**Authors:** Luzan Koster, Henk Nies

**Affiliations:** ^1^ Department of Organization Sciences Faculty of Social Sciences VU University Amsterdam The Netherlands; ^2^ City of Amsterdam, Dep. Mobility & Public Space Amsterdam The Netherlands; ^3^ Vilans National Centre of Expertise for Long‐term Care Utrecht The Netherlands

**Keywords:** family participation, identity, qualitative research, relationship‐centred care, residential dementia care, roles rights and responsibilities, triad encounters, user involvement

## Abstract

**Background:**

Researchers often stress the necessity and challenge of integrating the positionings of residents, family members and nurses in order to realize each actor's involvement in long‐term dementia care. Yet most studies approach user and family involvement separately.

**Aim:**

To explain how productive involvement in care provision is accomplished in triadic relationships between residents, family members and nurses.

**Methods:**

An ethnographic study of identity work, conducted between 2014 and 2016 in a Dutch nursing home.

**Findings:**

We identify four ideal‐typical identity positionings performed by nurses through daily activities. The findings reveal how their identity positionings were inseparable from those of the residents and family members as they formed triads. Congruent, or ‘matching’, identity positionings set the stage for productive involvement. Our systematic analysis of participants' identity work shows how—through embedded rights and responsibilities—their positionings inherently shaped and formed the triadic types and degrees of involvement observed within these relationships.

**Discussion and conclusion:**

This study both unravels and juxtaposes the interrelatedness of, and differences between, the concepts of user and family involvement. Accordingly, our findings display how residents, family members and nurses—while continuously entangled in triadic relationships—can use their identity positionings to accomplish a variety of involvement activities. To mirror and optimize the implementation of user and family involvement, we propose a rights‐based and relational framework based on our findings.

**Patient or public contribution:**

Conversations with and observations of residents; feedback session with the Clients' Council.

## 1 INTRODUCTION

Over the past four decades, long‐term care organizations, policymakers and scholars have increasingly promoted the involvement of both users and their family members in delivering and organizing care services. When the users' identity, as reflected in their life history and preferences, is taken as a starting point for good care,^1^ user involvement becomes crucial. However, some parties caution against overly prioritizing users' positionings, warning that the interdependencies and contributions of other actors, such as family and staff, may be overlooked.^2^ Particularly in nursing homes—where family members often advocate for residents' positionings, provide socio‐emotional support and assist in the continuation of ‘normal routines’^2^, ^3^—family involvement is often seen as the best guarantee of residents' wellbeing^4^ and integral to their quality of life.^5^ Given the increasing number of people with dementia, the extent to and ways in which family members and/or the users themselves (in this paper, ‘user’ refers to residents in the nursing home) should and can be involved in care provision is frequently discussed.^6^ Emphasizing the triadic relationship between residents, family and nurses, today's challenge lies in ‘how to involve and support a diversity of individuals, in ways that allow them to work [and live] in partnership’.^7^ ^(p. 626)^

Previous research has analysed the meaning and experiences of actors' involvement,^8^, ^9^, ^10^ interventions,^11^, ^12^, ^13^, ^14^, ^15^ the roles of family members and staff^3^, ^16^, ^17^—for example, visitor, mentor, helper or spokesperson—and their dyadic relationships^2^, ^5^, ^18^—for example, responsive, reciprocal, conflictive or collaborative. Such studies show how both parties frame their rights and responsibilities in involvement activities differently.^5^, ^19^ The importance of the staff's relationship with residents/ family members has also been well explained, for example, through trust and respect, open communication and information‐sharing, collaboration, the acknowledgement and reciprocity of needs, and a supportive environment.^2^, ^4^, ^20^, ^21^ However, research has usually focused on dyadic involvement of either users or family members with nurses, rather than on their involvement in triadic relationships (with some exceptions, eg^22^, ^23^, ^24^). Johnson et al^24^ ^(p.1305)^ have recently called attention to ‘a gap [in scholarship] concerning how the care relationship between older patients, their relatives and nurses in triad encounters is established’.

To understand the dynamics of their different positionings, we must understand what occurs when residents, family members and nurses meet and form a triadic relationship. Because their interactions—as manifested in care routines—shape involvement,^23^ we aimed to generate insights into how these actors pursue productive involvement in their triadic relationships on a day‐to‐day basis. Despite a lack of conceptualization,^25^, ^26^, ^27^ scholars commonly assume both user and family involvement to be rights‐based, democratic processes of equal citizenship.^5^, ^7^, ^28^ Building on this premise, we define involvement in nursing homes as a partnership between residents, family members and nurses (from assistants to head nurses) in which they use activities—such as engaging in social and personal experiences, dialogue, decision‐making and reciprocal support—to divide rights and responsibilities in order to influence, shape or contribute to daily care and residential life. Drawing from our ethnographic study of a Dutch nursing home, we propose a framework of identity work in situated interactions to analyse involvement activities. Before presenting both our findings and contributions, we will first elaborate on our framework's underlying theoretical assumptions.

## 2 IDENTITY WORK AND IDENTITY POSITIONINGS

Given a large body of work on rights‐based involvement that stresses the importance of roles and relationships in everyday encounters, we deemed a theoretical framework of situated interactional identity work to be particularly valuable. Indeed, people identify and enact their rights and responsibilities in care routines through their corresponding identities. In organizational settings, identity work implies that people do not passively undergo influences from others—for example, from management, their peers, competitors or clients—but rather they actively ‘work on’ their identities^29^ as they manage their everyday professional tasks and organizational requirements. People construct their identities through processes of social interaction as they seek to answer identity‐related questions, for example: ‘”Who am I?” or “How shall I relate to others?”’.^30^ ^(p.21)^

In this paper, the term ‘identity positionings’ refers to the sayings and doings used by individuals as they mutually and reiteratively position themselves and others in order to enact who they are within situated interactions.^31^ This definition highlights two useful aspects of identity work. First, it emphasizes the interactional dynamics, which suggest the dialogical nature^32^ of identity as ‘our understanding of who we are and of who other people are, and, reciprocally, [as] other people's understanding[s] of themselves and of others (which includes us)’.^33^ ^(p. 18)^ In other words, individuals not only position themselves, they are also positioned by others. In response, individuals may, for example, reaffirm their own positioning of the self,^34^ adjust the self to a group^35^ or contrast the self to and reject others' positionings.^33^ Second, by recognizing the inseparableness of ‘being’ and ‘doing’,^36^ situational dynamics address identity as it emerges in moment‐to‐moment organizational activity^37^—which helps explain how identities shift and vary from one situation to another.^38^ For instance, to simultaneously construct collaborative *and* competitive identities,^39^ a professional may navigate between, on the one hand, belonging to a social group and, on the other hand, a sense of uniqueness.^40^

Altogether, by focusing on situated and interactional dynamics, our conceptual framework helps further research on how involvement activities are accomplished through the triadic relationships of residents, family members and nurses as they interact during care practices and enact their setting‐specific identity positionings.

## 3 DESIGN AND METHODS

The implementation of a Dutch nursing home's ‘family participation policy’ prompted our ethnographic study on how identities and involvement are shaped in daily practice. We chose an ethnographic approach because it is commonly used to observe and trace the meanings and (inter)actions experienced by various actors in real time^41^, ^42^, ^43^—in our case, in everyday residential life and care practices.

### 3.1 Ethical procedures

Discussed in advance via written communication with the Ethical Review Commission of the university's faculty, all data have been processed according to the guidelines of the Dutch Central Commission on Research Involving Human Subjects for non‐medical research. The residence facility's management and ethical commission also gave their consent for this research and, on various occasions and through several channels, communicated the purpose of the research on an organizational level—for example, in their monthly newsletters for residents and family, in nurses' team meetings and in the Clients' Council. Moreover, before engaging in conversations with the fieldworker, both nurses and family members verbally consented to observation of themselves and the residents. The fieldworker also respected residents' expressions of welcoming or opposing her presence. To anonymize the data, all names in this article are pseudonyms.

### 3.2 Research setting

Seen as a frontrunner in the implementation of their family participation policy, the gated, long‐term care facility was selected as an ‘atypical case’ of involvement, which amplified the processes of analytical interest.^44^ Furthermore, because the facility had recently started to only admit residents with dementia, the urgency of family involvement was increased. Also of interest was the residence's ambition to emulate a ‘normal living situation’ through small‐scale housing facilities, which intensified staff encounters with family within the home. The building, dating from 2011, comprised six subunits, each with the archetypical features of a home, for example, its own entrance, living room, kitchen, shared bathrooms and private bedrooms. Used for group gatherings and entertainment events, the residence also had one central lobby, a garden and a hotel‐like lounge.

### 3.3 Data collection

Most data were collected between August 2014 and August 2016, encompassing 325 hours spent in the residence and almost 700 pages of fieldnotes. In the first 3 months, the fieldworker shadowed^45^ ten nurses. During morning, evening and night shifts on weekdays and in weekends, she observed their interactions with numerous residents, their relatives (including friends and neighbours), other nursing home staff (from nurse assistants to head nurses), medical specialists and managers. These interactions included the naturally occurring ‘sayings and doings’ encountered in the home's central spaces, the residents' shared living space and—by exception and only when invited by residents or staff—in residents' own private spaces. For approximately the first year, the fieldworker volunteered once a week as a cook for residents and participated in a wide‐range of events with the theme of family participation (eg family meetings and dinners, employees' team‐building day, management's vision day). In the second year, she continued to track family participation‐related events and volunteered as a chaperone on day trips. Throughout 2017 and 2018, she kept in contact with her key informants, gratefully receiving updates.

As she collected data, the fieldworker questioned participants to avoid misinterpreting their sayings and doings^43^ (see Appendix 1). Becoming increasingly familiar with and to all participants, she stepped out of the field multiple times to reflect on her data through writing and discussions with her co‐author, who remained out of the field in order to assure an outsider's perspective.^41^ To interpret the findings, feedback sessions were held with the facility's management, Board of Directors and Clients' Council, Employees Council and Research Commission.

### 3.4 Data analysis

Our analytical procedure comprised an inductive, iterative process.^42^, ^46^ Having initially discussed fieldnotes with a focus on how nurses handled family participation, we first identified and compiled nurses' identity positionings. By going back and forth between the data and literature, the themes of ‘everyday care routines’ and ‘dialogue’ emerged (later denoted as situated and interactional dynamics), which revealed the entanglement of triadic relationships. Accordingly, we also analysed residents' and family's identity positionings.

Next, by ‘zooming in and out’ to compare the observed involvement activities to theoretical conceptualizations,^47^ the theme of ‘rights and responsibilities’^5^, ^7^, ^28^ was developed. The notion that people locate rights and responsibilities based on their setting‐specific identities led to further comparison and the refinement^48^ of ‘identity positionings in triads’ in correspondence with ‘types and degrees of involvement’. Finally, being interested in productive involvement, we focused on the three parties' congruent or ‘matching’ identity positionings as reflected in the situated interactions we deemed positive or harmonious. Our analysis of the conflicting identity positionings and their inseparable involvement activities is presented elsewhere.

## 4 FINDINGS

In this section, we describe the predominantly performed identity positionings of residents, family members and nurses. Our findings elaborate on these actors' productive triadic formations—each of which concerned a different type and degree of triadic involvement—and on how these actors divided their corresponding rights and responsibilities.

### 4.1 Triads: identity positionings of residents, family and nurses

Because the positioning of one's self inherently implies the positioning of others, residents, family members and nurses inevitably came to form specific constellations or ‘triads’. In harmonious situations and interactions, the triads comprised matching identity positionings, which we define as the sayings and doings through which these parties mirrored, confirmed or reinforced each other's identity positionings. In this section, we describe the four triads we observed in relation to residents' and family members' most frequently occurring positionings vis‐à‐vis nurses.

First, residents acted like *care recipients* by screaming, crying, moaning or grimacing in pain, for example: ‘It's so cold in here!’ (Mrs Biddle); and, ‘Nurse! Where are my pills?’ (Mrs David). Such interactions forced nurses to take on their role of *care expert*. At times, we heard residents invoke their family members as *experts of experience* on their behalf: ‘I don't know which medicines I take, ask my son’. Second, many residents expressed feeling like *vulnerable persons*: ‘It's better that I'm here, you know. I'm better than most here, but sometimes I get confused. Then I do funny stuff. [My daughter] didn't know what to do. She cried’ (Mrs Logan). This quote illustrates residents' recognition of their family as *care recipients* who need nurses' *shoulders* for socio‐emotional support and to take over caretaking tasks. Third, residents positioned themselves as *homeowners*, for example: ‘This is my home now, I'm grateful. I have a roof over my head, a nice bedroom, I eat until I'm full. My family comes to visit me here’ (Mrs Hall). Likewise, residents searched for *social companionship* from their nurses and family members, for example, by chatting, winking or adopting a social role: ‘I'm the joker of the house!’ (Mrs Little). Fourth, residents also sometimes acted like *relinquishers* when they did not mind waiting or adjusting to the rhythm of nurses (and sometimes their helping family) as they organized things, for example: ‘Dinner will be ready soon – first [the nurse] has other things to do”; and, “My daughter is cleaning my room, I'll wait for her here’.

Family members, who—first—also considered themselves to be *experts of experience*, were able to augment nurses' care expertise thanks to their knowledge of residents' character traits, preferences, habits, and prior life and care experiences. ‘She used to get locked up in the broom closet as a kid. The nurses didn't understand why she was screaming every night, so I told them. Now they leave her bedroom door open!’ (Julie). Second, family members would position themselves as *extended care recipients,* leaving the nurses to look after *the vulnerable older persons,* and seeking nurses' compassion; hence our identification of nurses' positioning as *shoulders*. As one granddaughter explained: ‘My mom took care of my grandma when she still lived at home, but she doesn't come here. She can't bear to see her mother this way! She's relieved that the nurses are taking care of her now’. Third, family members also often related to the residents as *companions* while viewing the nurses as *socializers*, for example: ‘Mom and I just watch TV or have coffee. Today I baked a cake. There's a piece for everyone. [Nurse] Alice, will you grab some plates? Let's make it nice in here!’ (Holly). Fourth, family members also behaved as the busy nurses' *helpers* who felt impelled to organize housekeeping: ‘I always do the laundry, that's one less thing for the nurses to do’!

By framing residents as *care recipients*, nurses—first and foremost—positioned themselves as *care experts* who fostered intimate relationships and provided personal attention in order to both get to know each resident and to handle disruptive or delicate situations, for example, bathroom needs or public undressing. As such, and by gathering information from family, nurses conceived family as *experts by experience*, for example: ‘Mrs Costa's son told me she's not a morning person so, before bringing her into the living room, I always gently open the curtains and give her breakfast in bed or sit down with her and chat a little’ (nurse Chloe). Second, nurses would serve as compassionate *shoulders* to lean and cry on for the family of *vulnerable persons,* who were viewed as *extended care recipients*: ‘You know, most of them just went through a heavy process of losing their loved one as they knew them, who they already gave as much care as they could’ (nurse Jaimè). Third, nurses also acted like *socializers* by greeting, making conversation, joking and focusing on social activities: ‘Sometimes I put on some music or the TV, or paint their nails; they enjoy that’ (nurse Abigail). Likewise, nurses often included family members, for example, by playing a game or dining together: ‘You're all part of the family, because this is [the residents'] home now and you're all together so much’ (nurse Bob). In this manner, nurses positioned the residents as *homeowners* and their visiting family as their *companions*. Finally, nurses often positioned themselves as *organizers*, which meant formulating and sharing information about residents with colleagues, as well as organizing the household (cleaning, cooking, dating opened food packages, etc): ‘It's my job to provide a safe and clean environment’ (nurse Olivia). For this purpose, nurses expected family to act as *helpers* and residents as *relinquishers* to their work routines.

**Figure 1:**
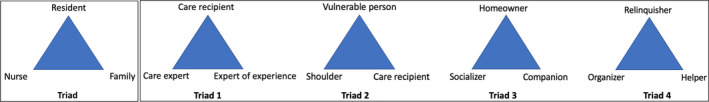
Four triads in harmonious situated interactions: the matching identity positionings of residents, family members and nurses

In short, by positioning themselves, each party also positioned the others, either explicitly or implicitly. Consequently, the residents, family members and nurses formed triads. Figure 1 displays the four triads as described above, which determined their matching identity positionings.

### 4.2 Manifestations of involvement: rights and responsibilities

A rights‐based approach, on which our analysis is based, grounds the involvement activities we observed in the rights and responsibilities of all actors. By identifying whose and which rights and responsibilities were embedded in each of the four observed triads of identity positionings, this section places the underlying types and degrees of triadic involvement on a continuum from mostly cooperative (ie contributions resulting from either non‐action or others' initiatives) to mostly operational (ie contributions resulting from self‐initiation).

#### 4.2.1 Triad 1

The right of residents to receive professional, tailored care: this focus motivated nurses to learn and execute the residents' care preferences and needs—for example, by preparing their favourite foods, bringing them to bed at the time of their preference or helping them get dressed in a specific way. Nurses felt responsible, explaining: ‘You have to really understand the person. They often can't just tell you. You have to put yourself in their shoes and think: what do they want? Why do they do what they do?’ (nurse Graciela). Nurses also welcomed insights from family members, who were seen as experts of residents' past preferences, habits and care needs: ‘When family members join for dinner, we learn more about the residents and their past. That really helps us understand them better’. The families echoed this sentiment: ‘We create “life‐story books” so the nurses can learn who they are and what they want’. In other words, nurses aimed to enact *perceived user involvement*—a term we suggest to mean the hearing and voicing of residents' care perspectives based on the caregiver's own sensory observations and inquiries regarding the residents' preferences, needs and intentions (in context of this study, the words ‘user’ and ‘resident’ are interchangeable). In order to accomplish perceived user involvement, nurses also invoked the support of family. Because nurses did not expect the residents and their families to take responsibility for acting or speaking up, their degree of involvement is described as *cooperative*.

#### 4.2.2 Triad 2

The right of family members to be supported in dealing with the disabling condition of their loved ones: this imperative was underlined by both the nurses and the residents. Residents often considered their family's best interest more important than their own vulnerabilities. They effaced themselves to enable support for their family, for example: ‘I wanted to stay home, I really did. But my daughter cried. I don't want her to cry anymore. It's better this way' (Mrs Logan). Nurses took responsibility for care tasks, claiming it was too much for the family: ‘[Family members] don't bring their loved ones here for no reason. Now it's our turn’ (nurse Phil). Family members also emphasized the responsibility of ‘paid professionals’: ‘Because of you, we can breathe. I used to cry and cry all day long, but now I see my dad's okay. For the first time just now, he said he was home. He meant this is his home. That really helps me’ (Heather). As these quotes illustrate, *perceived family involvement*—that is, the hearing and voicing of family's views on both their own willingness to be involved and the residents' personal circumstances and care experiences—was a central focus that *cooperatively* involved residents and family alike.

#### 4.2.3 Triad 3

The right of residents and their family members to feel socially valuable and included in the home: this, nurses expressed, was also their responsibility. This was made evident by their continuous efforts to socially engage: ‘Mrs Alden [a resident] always helps me set the table. I like to sit down and eat with the residents (…). It's like back in the day in my dormitory, friends are always walking in and out’ (nurse Henry). Likewise, family members often volunteered to share the responsibility of encouraging residents and each other to participate in social activities: ‘[Nurse] Otto is always so good at making things fun, he's just great! For example, he started organizing theme dinners. Then others took his lead and we [as relatives] are now arranging dinners ourselves’ (Lilli). Residents also made themselves useful, but were not seen as responsible: ‘After dinner I always clear the table. But first I'll sit down. This is my seat, next to Mrs Penny, because it's my job to help her eat’ (Mrs Draper). This right implicates the entwinement of user and family involvement as nurses aimed to engage both residents and their family members, and family members sought to engage residents and other family. Because nurses and family members shared in this responsibility, but residents' efforts were seen as optional, the *social user and family involvement* of residents can be seen as *cooperative,* and that of family and nurses as *operational*.

#### 4.2.4 Triad 4

The right of nurses to be recognized for and supported in their workload. Residents often confirmed that nurses were busy: ‘I think the nurse is with Mrs Patrick, in the bathroom. I don't want to bother her, she has enough to worry about. I'm doing just fine, enjoying my tea’ (Mrs Steven). Similarly, nurses claimed that family members were also partly responsible for providing instrumental care, viewing them as a source of hands‐on assistance: ‘When [a family member] is here and I know everything is getting done, then I can use my time to tuck [residents] in after dinner. It's the only one‐on‐one time we have!’ (nurse Marijke). Family members also displayed a feeling of responsibility, especially regarding housekeeping: ‘Just tell me what needs to be done, I'm here to help. I can cook, but last time you preferred me to change the bed sheets’ (Kaitlyn). This type of involvement entails the instrumental assistance of nurses by family members. To unburden family and nursing staff, residents would sometimes refrain from asking for help, which enabled *instrumental family involvement*—which, accordingly, implied family's *operational* and, again, residents' *cooperative involvement*.

In sum, each party's triadic identity positioning determined the applicable rights and responsibilities of residents, family and nurses, which then shaped their types and degrees of involvement (see Table 1). Given a lack of ascribed responsibility for speaking up or taking action, residents' involvement emerged in cooperative degrees and in forms of ‘perceived’ and ‘social’ user involvement. By abstaining from voicing their own needs in order to support and unburden family and staff, residents also played a cooperative role in family involvement. For their part, family members were typically cooperative—that is, unobligated—in regard to ‘perceived’ user and ‘perceived’ family involvement, but more operational when it came to ‘social’ and ‘instrumental’ family involvement. Nurses, in contrast, always took responsibility for the rights of others and were therefore always operationally involved.

**Table 1:** Triadic involvement: From matching triadic identity positionings to types and degrees of triadic involvement

Identity positionings 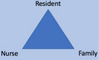	Triad 1 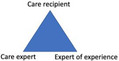	Triad 2 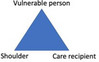	Triad 3 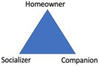	Triad 4 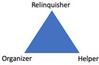
Whose rights are central?	Residents	Family	Residents and family	Nurses
Whose responsibilitiesare central?	Nurses	Nurses	Nurses and family	Nurses and family
Type	Perceived user involvement	Perceived family involvement	Social user and family involvement	Instrumental family involvement
Involvement of residents	Voicing and being heard regarding personal care perspectives	Effacing one's own interests to support family	Participating in social and daily activities in order to be included in residential life	Withholding care demands to enable family and staff to carry out practical tasks
Involvement of family	Providing information to support residents' autonomy vis‐à‐vis personal care decisions	Voicing and being heard regarding one's own experiences with residents and their care histories	Participating in social and personal activities in order to be included in residential life	Providing instrumental assistance to lighten caretaking tasks
Involvement of nurses	Gaining information on residents' care perspectives in order to include them in daily decisions	Gaining information on family's perspectives on and experiences with residents' care in order to support family	Encouraging social and personal activities in order to include residents and family in residential life	Pragmatically organizing one's own workload in order to provide a structured, safe and hygienic environment
Degree				
Involvement of residents	Cooperative	Cooperative	Cooperative	Cooperative
Involvement of family	Cooperative	Cooperative	Operational	Operational
Involvement of nurses	Operational	Operational	Operational	Operational

## 5 DISCUSSION

Most related studies focus on the dyadic relationship between either staff and users or staff and family members, as opposed to the triadic relationship of all three. According to our findings, because each party encounters the rights and responsibilities of both their own and the others' identity positionings, different types and degrees of involvement can best be explained in the context of triads. When the residents, family and nurses were able to congruently divide their rights and responsibilities, their triads reflected productive triadic involvement. Drawing attention to the idiosyncrasies of triadic involvement, then, also reveals the interrelatedness and differences of both user and family involvement, and its micro‐interactional nature in the context of long‐term dementia care.

First, since partnership‐building efforts with residents and family simultaneously develop and affect all parties, our findings demonstrate that separately approaching user and family involvement is insufficient. Indeed, to ensure perceived and social user involvement, nurses involve family through advocacy, social inclusion and validation. Reflecting the reciprocal nature of family involvement, we also observed—in cases of perceived and social family involvement—residents' effacement of their own needs in favour of their family's interests. Similarly, by supporting and welcoming their presence, family felt encouraged to accomplish both user and family involvement: family members' efforts helped nurses create more opportunities to better support residents, for example, by freeing up time for them to give each resident more personal attention. In this sense, user and family involvement formed two sides of the same coin—a finding supported by similar studies.^23^, ^49^, ^50^, ^51^, ^52^ Additionally, our framework shows *how* inherently related identity positionings can enable the involvement of both users and family: when the established triadic identity positionings ‘match’, the resulting agreements vis‐à‐vis rights and responsibilities serve to guide and define the (potential) involvement of each party.

Second, our framework helps unravel the differences between user and family involvement in a triadic context. Johnsson et al^24^ have already begun to explain how triadic relationships between residents, family and nurses may manifest in practice: first, by using the pragmatics of care routines to start a conversation, then by the presentation of ‘niche information’ from each party, and finally by addressing and integrating each party's perspective to form a new, agreed‐upon view. While this model accounts for generic contributions and responsibilities, our study identifies which specific positionings of residents, family and nurses triggered which specific types and degrees of involvement. These distinctions are valuable because they correspond to different purposes and practices^28^—including each actor's diverse rights and responsibilities. For example, ambitions to safeguard residents' personal autonomy (eg regarding when and how often a resident showers) require acts of soliciting, respecting and advocating for residents' past and present care perspectives,^6^, ^26^ and such acts require the involved parties to form a triad: the care recipient/care expert/expert of experience triad. Other ambitions may prioritize the unburdening of family (eg responding to overwhelmed family members). This leads to acts of listening and support, which trigger another triad: the vulnerable person/shoulder/care recipient triad. Awareness of these differences may help actors navigate and balance the other parties' different positionings, which may in turn help them identify which involvement activities are best suited to which situated interactions.

These findings respond to calls for research on the scope, extent and nature of user and family involvement^25^ in specific care settings.^53^ Researchers have identified involvement in different areas—such as in the delivery, development, planning, evaluation and recruitment of services^26^, ^28^—and at different levels—such as in societal policy‐making, collective advocacy groups, on a service or organizational level, and in individual or direct care.^7^, ^26^ As involvement for residents with dementia implies the right to experience productive service delivery and to express themselves through both verbal and body language,^27^, ^50^, ^54^ it is particularly important for residential facilities to scrutinize involvement at a micro‐level. Additionally, we have placed involvement on a continuum from mostly cooperative to mostly operational: operational meaning to take responsibility and resulting from self‐initiation; cooperative meaning to act on others' initiatives without being responsible or to purposefully refrain from acting, for example, by not asking for help. This is in line with Tritter's^28^ ^(p. 267‐277)^ distinction between the indirect involvement of service users, for example, by informing caregivers of their preferences, and their direct involvement, for example, by taking part in actual decision‐making. In the context of this paper, we considered ‘actual decision‐making’ as it applied to everyday issues, such as breakfast, schedules or music preferences. Indeed, our findings indicate that the nature of involvement in long‐term dementia care concerns small, direct and personal gestures rather than big, overarching activities.

Often citing Arnstein's^55^ ladder, researchers have warned for tokenism: if people do not have the ‘muscles’ to enforce their views, then allowing them to have a voice and be heard—that is, involvement—is only symbolic. In such cases, critics have questioned whether involvement should be claimed as actual involvement.^56^ Others^7^, ^54^, ^57^ have been concerned with the concept of self‐control, which may overly rely on both performative identities and activities that require more advanced communication skills and decision‐making capabilities. These potentially exclude those who do not possess such competencies, for example, people with dementia. Nevertheless, despite their more powerful status, professional caregivers do have goals (guaranteeing residents' wellbeing, executing care routines, etc) for which they depend on others in the triad.^58^ This implies that the power of professionals does not invariably diminish the power of others, but rather that others possess different kinds of power that are both triggered by and produced through the dynamics of a triad.^28^, ^59^ Resonating with our data, we follow these notions to argue that residents can partake in involvement by expressing their care experiences, all the while taking others into account—for example, by supporting or respecting them (or, negatively, by acting obstructively or making demands that hinder the actions of others). In order to overcome stigma and tokenism, it is crucial to account for a varied range of strengths and skills, and to offer appropriate, creative, diverse and structural opportunities for triadic involvement.^5^, ^7^, ^49^

## 6 LIMITATIONS

Our study has aimed to explain productive user and family involvement by developing a framework that captures actors' identity positionings in triads. Consequently, our study does not contain an exhaustive overview, but rather an in‐depth look at the prototypical identities enacted within a residential care facility. According to our field observations, participants in this study did not necessarily engage in each triad (eg not all family members positioned themselves as ‘helpers’, nor did all residents behave as ‘homeowners’). Also, within each category of actors, different persons preferred different identity positionings (eg nurse Otto was more of a ‘socializer’, while nurse Graciela was more of a ‘shoulder’). These positionings sometimes overlapped (eg when family offered help by assisting residents during family dinners). Instead of comprehensively elaborating on the characteristics and preferences of each identity positioning as researchers have already done extensively, we focused on the consequences of each positioning in terms of its types and degrees of involvement in a triadic context.

Moreover, not all situated interactions proved harmonious. While we have elsewhere analysed how tensions arise as a result of incongruent triadic identity positionings, such interactions were beyond the scope of this paper. Given the complex power inequalities that emerge in the identity work of residents versus family and nurses, we hope that future research will further explore both the implications of identity work and power in the context of people with dementia, and how the involved actors negotiate and achieve shared solutions. Indeed, we acknowledge that there is a fine line to walk—between over‐estimation and under‐stimulation—when it comes to safeguarding the ability of people with dementia to meaningfully partake in involvement.

Furthermore, and in contrast to most care settings, nursing homes for people with dementia require relatively long‐standing and interdependent relationships. Actors' identity positionings may therefore evolve over time and as the conditions of the residents, family, nurses and/or the care organization change. Whereas we primarily shadowed nurses, future research may want to focus more on the changing networks around such residents and their family members.

## 7 CONCLUSIONS

Our framework contributes to a better understanding of triadic and interdependent relationships, which may also be applicable to other long‐term care services, for example, those for people with chronic physical disabilities, psychiatric conditions or child care. More specifically, our framework may help both researchers and practitioners examine which identity positionings influence the legitimacy and acceptance of triadic involvement and through which embedded rights and responsibilities. As was done in the residence facility we studied, training and counselling programmes for nurses and family may then be developed to address specific complex situated interactions. To compile a diversified team, managers may choose to profile and recruit nurses according to their competencies vis‐à‐vis different types and degrees of involvement.

In order to facilitate destigmatized, equal and inclusive involvement in the context of long‐term dementia care, our findings suggest that researchers and practitioners must reframe the concept and practice of triadic involvement as a dynamic, relational micro‐endeavour: through a lens of identity work and with a focus on amplified opportunities for, and contributions from, people with dementia together with family and nurses. Involvement in dementia care, in sum, is a large concept practised through small, subtle and situated (inter)actions.

## CONFLICT OF INTEREST

The authors declare that there is no conflict of interest.

## AUTHOR CONTRIBUTIONS

All data were collected by the first author. Both authors have made substantial contributions to the present study's conception, data analysis and interpretation. The first author drafted the manuscript and both authors revised it critically for important intellectual content. Both authors have approved the final version to be published and agreed to be accountable for all aspects of the work.

## ETHICAL APPROVAL

All data have been processed according to the guidelines of the Dutch national Central Commission on Research Involving Human Subjects (CCMO) for non‐medical research (WMO) and based on written communication with the Ethical Review Commission of the university's faculty. The residence facility's management and ethical commission gave their consent for this research and, various times and through several channels, communicated the purpose of the research on an organizational level for example, in their monthly newsletters for residents and family, in nurses' team meetings and in the Clients' Council. Moreover, before engaging in conversations with the fieldworker, participants gave their oral consent. To anonymize the data, all names used in this article are pseudonyms.

## Data Availability

The data that support the findings of the present study are available on request from the corresponding author. The data are not publicly available due to privacy and/or ethical restrictions.

## APPENDIX 1

### TOPIC GUIDE FOR INFORMAL QUESTIONING

Throughout our collection of data, the fieldworker used the following guide to informally question and probe all participants (eg while shadowing, during participant observations or casual conversation). She aimed to follow participants' terminology, and to signal additional topics and issues raised by participants.

**Table:**

Participants	Topics
Nurses Family members Managers Medical specialists	According to you… Re: nurses What makes a ‘good’ nurse? What qualities and goals do good nurses have? What are nurses' tasks within the residence? Do nurses involve family and—if so—how? Re: family How do you view the role of family members within the residence? What are their tasks? How do nurses and family members communicate? How do nurses and family members collaborate? Re: residents What do residents consider important? What does ‘good’ care mean to them? How do you know what residents need or want? How can and should you involve residents? How do residents relate to or interact with nurses/family members? Re: policies With regard to the residence's new policies, what changes have you noticed in the way things are done? What could/should be improved?
Residents	During any given day, what do you do? How often and with whom do you…? Perform daily routines (eg shower, set the table); Participate in events (eg go outside, exercise, play games). How often do you see…? What do you (dis)like about…? How do you feel about…? Nurses; Your family; The home (eg being here, what and how things are done).
